# Concurrent Imitative Movement During Action Observation Facilitates Accuracy of Outcome Prediction in Less-Skilled Performers

**DOI:** 10.3389/fpsyg.2018.01262

**Published:** 2018-07-20

**Authors:** Satoshi Unenaka, Sachi Ikudome, Shiro Mori, Hiroki Nakamoto

**Affiliations:** ^1^Department of Sport Education, School of Lifelong Sport, Hokusho University, Ebetsu, Japan; ^2^Faculty of Physical Education, National Institute of Fitness and Sports in Kanoya, Kanoya, Japan

**Keywords:** outcome prediction, perceptual training, motor simulation, sports expertise, free throw

## Abstract

Skilled athletes can predict the outcome of actions performed by others, based on the kinematic information inherent in others’ actions, earlier and more accurately than less-skilled athletes. Activation of the motor cortex during action observation indicates motor simulation of other’s actions in one’s own motor system; this contributes to skilled outcome prediction. Thus, the present study investigated whether concurrent movements during action observation that affect motor simulation influence the accuracy of outcome prediction, namely, whether concurrent imitative movement and self-movement enhance and inhibit accuracy, respectively, based on skill level. Twelve male varsity basketball players (skilled group) and twelve male college students with no special training in basketball (less-skilled group) were required to predict the outcome of a basketball free throw by another player based on the action kinematics in the following four conditions: prediction without any action (observation), prediction with right-wrist volar flexion with maximum speed (incongruent-action), prediction with concurrent imitative movement during observation by right-wrist flexion as if imitating the model’s action (imitative-motion), or prediction with concurrent self-movement by right-wrist flexion as if shooting by oneself (self-motion). The results showed that the skilled group had degraded accuracy of outcome prediction in the self-motion condition compared to the observation condition. In contrast, accuracy in the less-skilled group was facilitated in the imitative-motion condition compared to the observation condition. The findings suggest that, at least in less-skilled participants, the appropriate motor simulation that relates to skilled prediction can be virtually induced by concurrent imitative movement during the prediction task, even if they have less experience of free throws. This effect in imitative movement is likely to occur by producing identical motor commands with observed action, thereby enabling the prediction of sensory consequences and outcome accurately via a forward model. We propose that traditional perceptual training with concurrent imitative movement is likely to be an effective way to develop visual- and motor-based hybrid outcome predictions that produce superior inferences in skilled athletes.

## Introduction

A well-known feature of skilled athletes is their superior ability to make predictions (e.g., Abernethy et al., 1999; Williams et al., 2003; Hagemann et al., 2006; Savelsbergh et al., 2010). In ball games, such as basketball and soccer, earlier and more precise prediction of future states that arise from opponents’ actions and/or the ball trajectory enables players to make more optimal and faster decisions in a dynamic environment. Indeed, the ability to make such predictions is a predictor of performance in real sports settings (Müller and Fadde, 2016). Therefore, the topic of how to improve prediction abilities has received attention for a considerable period in sports psychology and related domains.

Perceptual training has been proposed as an effective way of improving prediction abilities. The rationale of perceptual training is that skilled athletes who have superior prediction abilities can perceptually identify specific movement patterns inherent in opponents’ actions that are associated with a specific outcome (i.e., anticipatory kinematic cues; Abernethy, 1990; Tenenbaum et al., 1999; Jackson et al., 2006; Abernethy and Zawi, 2007; Williams et al., 2009). Regarding kinematic-cue utilization, a basic training method is for learners to repeatedly predict the outcome of an opponent’s actions (e.g., serve direction *after* racket-ball contact in tennis) in videos (i.e., a sports-specific scene that is filmed from a player perspective) that are occluded at various time points; after the prediction is made, the correct answer is provided as feedback. Thereby, learners develop associations between anticipatory kinematic cues and outcomes through intensive visual exposures. Various types of training methods have been tested recently, such as guided discovery and/or gaze cueing based on advance cue utilization; these have confirmed the effectiveness of perceptual training (e.g., Abernethy et al., 1999; Williams et al., 2003; Jackson and Farrow, 2005; Hagemann et al., 2006; Savelsbergh et al., 2010; Hopwood et al., 2011; Ryu et al., 2013).

In contrast, although learners accumulate knowledge about kinematic-outcome associations during perceptual training via *perceptual experience*, several recent studies have indicated the importance of *motor experience* for enhancing prediction abilities of athletes (Aglioti et al., 2008; Cañal-Bruland et al., 2012; Urgesi et al., 2012; Tomeo et al., 2013; Ikegami and Ganesh, 2014; Mulligan and Hodges, 2014; Makris and Urgesi, 2015; Mulligan et al., 2016a,b; Denis et al., 2017). Aglioti et al. (2008) reported that elite basketball players, who have considerable motor experience, could predict the shot outcomes of others based on the shooter’s throwing kinematics more accurately than individuals with considerable visual experience (coaches or sports journalists) and novices (see also, Cañal-Bruland et al., 2012; Urgesi et al., 2012). In addition, Mulligan and Hodges (2014) found that outcome prediction in dart throwing was improved by motor learning of dart throwing itself, even when all visual information (e.g., own actions and dart trajectory after throwing) was completely excluded during learning. That is, skilled prediction is not developed merely via perceptual experience. Furthermore, recent evidence suggests that perceptual and motor experience develop different prediction mechanisms (Aglioti et al., 2008; Urgesi et al., 2012; Makris and Urgesi, 2015), namely visual- and motor-based prediction, respectively (Mulligan et al., 2016b). Motor experience more greatly improves prediction abilities based on kinematic cues than does perceptual experience such as observation of other’s actions (Urgesi et al., 2012). Therefore, additional focus on the role of motor experience is needed to develop effective training of prediction abilities; however, few studies have focused on motor experience as compared to those that have considered perceptual experience.

The influence of motor development on perceptual predictions is consistent with the notion of bidirectional links between perceptual (observation of other’s actions) and motor (execution of one’s own actions) representations. It has been proposed that the perceptual and motor systems partly share the same representations (Prinz, 1990, 1997; Schütz-Bosbach and Prinz, 2007). Therefore, development of motor representations also affects the perception of the same action performed by others. Consistent with this notion, neuroscientific studies have shown that during prediction tasks, skilled athletes display enhanced activity of neural networks, including frontal, parietal, and temporal regions of the brain (Wright and Jackson, 2007; Aglioti et al., 2008; Abreu et al., 2012; Wu et al., 2013). These are activated both when executing one’s own actions and while observing other’s actions; the latter is referred to as the action-observation network (AON) and/or mirror neuron system (Gallese et al., 1996; Rizzolatti and Craighero, 2004; Iacoboni et al., 2005). Therefore, it has been proposed that motor activation during action observation contributes to the superior prediction abilities of skilled athletes (Aglioti et al., 2008; Abreu et al., 2012; Makris and Urgesi, 2015).

Interestingly, some studies have suggested that activation of the motor system during action observation changes depending on how observers monitor others’ actions (i.e., the observers’ intentions). Buccino et al. (2004) reported that neural activity during action observation is facilitated, including in the motor area, when participants observe with the intention of imitation. Additionally, motor activation during action observation can be facilitated through sensorimotor learning that has temporal and spatial congruencies between observed and executed behaviors (i.e., imitation; Catmur et al., 2007; Vogt et al., 2007; Heyes, 2010; Ménoret et al., 2013). In contrast, if self-focus is present, which may be elicited by engaging participants in a self-referential task before action observation, the motor activation that relates to the imitation is inhibited (Spengler et al., 2010). Additionally, observation of actions attributed to another agent facilitates motor-system activity, whereas observation of identical actions linked to the self does not (Schütz-Bosbach et al., 2006). That is, if others’ actions are viewed with the intention of imitation (i.e., imitative movement), then action perception is facilitated.

In addition, Christensen et al. (2011) reported that when observers attempted to execute arm movements that were temporally and spatially congruent with those of an observed actor, the observer could accurately recognize the specific arm movement executed by actor, although they did not assume the effect of conscious cognitive processes such as intention. Nevertheless, this suggests that motor-based prediction might be changed by intention and similarity between observed and executed movement during action observation. More specifically, if an observer executes concurrent imitative movements, prediction accuracy is improved, because imitative movement is likely to facilitate spatial and temporal congruency with observed movement (Christensen et al., 2011; Springer et al., 2011) and enhance motor activation (Buccino et al., 2004). Further, if an observer executes concurrent self-focused movements, then prediction accuracy is degraded, because it would degrade congruency with observed movement and inhibit motor activation (Spengler et al., 2010).

Moreover, these effects come from concurrent imitative and self-focused movement (i.e., activate/inhibit motor simulation) and would be modulated depending on the skill level. As mentioned above, skilled athletes use motor simulation for predicting the action outcome of others. Therefore, no additional effect of imitative movement would be seen for skilled athletes in terms of prediction accuracy, because they already use a simulative process that would be induced by observing the action. In contrast, prediction accuracy would be degraded through concurrent self-focused movement because it would inhibit motor simulation processes in progress during the prediction task. On the other hand, for less-skilled people, imitative movement may facilitate prediction accuracy. Abreu et al. (2012) reported that activity of neural networks, including frontal, parietal, and temporal regions of the brain (AON network) were also activated in novices, although they demonstrate lower prediction ability. This implies that even novices engage in motor simulation during prediction tasks. It is believed that motor simulation enhances prediction accuracy according to the internal forward model, which enables us to predict future sensory consequences and outcomes based on an efference copy of issued motor commands (e.g., Mulligan and Hodges, 2014; Mulligan et al., 2016a,b). From the above evidence, it can be considered that novice and/or less-skilled people who have less motor experience can use motor simulation but that their motor commands created through observation are not likely to be accurate because they are less-developed. Therefore, the forward model would not produce appropriate predicted sensory consequences and outcomes. In other words, if individuals can produce the accurate motor commands during observation, then they can estimate the action outcomes correctly via the forward model. Taking these considerations into account, it may be that, in less-skilled individuals, concurrent imitative movement during action observation enhances the production of appropriate motor commands; thereby prediction accuracy will be temporarily improved. In contrast, concurrent self-focused movement in less-skilled individuals will not affect prediction accuracy if the motor simulation process is inhibited, because there was no reliance on motor-based prediction processes (Aglioti et al., 2008).

Thus, the purpose of the current study was to investigate how prediction accuracy is influenced by concurrent motor execution with different movement types during action observation. Accordingly, we recruited skilled basketball players, who were experts in motor-based outcome prediction (Aglioti et al., 2008; Abreu et al., 2012), and less-skilled players, who did not have such a prediction capability (e.g., Mulligan et al., 2016a). The occlusion technique was used to assess outcome-prediction capabilities: the participants made predictions about ball-landing locations near the hoop based on the actions of a model who performed basketball free throws. The task consisted of four conditions: observation without action, incongruent-action, imitative-motion, and self-motion. The observation condition was used to assess the baseline of prediction ability of each participant and to confirm the presence of skill-related differences in prediction ability. The incongruent-action condition was used to verify that the skilled athletes used motor-based predictions in the present study. Previous studies have demonstrated that incongruent actions degrade prediction accuracy in observers who use motor-based predictions, but not in observers who do not have such a capability (Mulligan et al., 2016a,b). Therefore, if skilled participants in the present study had motor-based prediction abilities, then their prediction accuracy would degrade, whereas if less-skilled participants did not have well-developed motor-based prediction abilities, then their prediction accuracy would be unaffected by their execution of incongruent actions. We hypothesized that prediction accuracy would be modulated by imitative-motion and by self-motion. Further, we hypothesized that these effects would vary, depending on the initial prediction ability (i.e., motor-based prediction ability).

## Materials and Methods

### Participants

Twelve male basketball players (skilled group; *M* = 20.4 years, *SD* = 1.7) and 12 male varsity students (less-skilled group; *M* = 23.9 years, *SD* = 2.1) participated in this study. All participants had normal or corrected-to-normal visual acuity in both eyes and always used their right hand to shoot a basketball. The skilled group had been playing competitive basketball for 8–13 years (*M* = 10.8 years, *SD* = 1.7 years). The less-skilled group had experience in playing basketball in physical education class, but no members of this group had experienced systematized training and competitive activities for basketball. This study was approved by the Ethics Committee of the National Institute of Fitness and Sports in Kanoya and was consistent with the institutional ethical requirements for human experimentation in accordance with the Declaration of Helsinki. Prior to the measurement session, all participants were fully informed of the procedures and possible risks, as well as the purpose of the study, and their written informed consent was obtained.

### Stimuli

To create occlusion video clips for this experiment, basketball free throws performed by a right-handed male basketball player who had 10 years of experience were digitally recorded using a hybrid camera (GC-PX1, JVC). The video camera was approximately 6 m from the player. A side-on perspective was recorded, such that the player and basketball hoop were visible. The player was requested to perform 50 trials each of three types of basketball free throws. First, the player performed prototypical moves in order to drop the ball through the hoop without touching it, that is, to successfully shoot (“in shot”). Second, the player altered the kinematics such that the trajectory of the ball fell short of the basketball hoop (“short shot”). Third, the player altered the kinematics such the trajectory of the ball went beyond the hoop (“long shot”). Additionally, the angles of the player’s right wrist were recorded by 3D motion analysis (NDI, OPTOTRAK Certus) during the shot release. Aglioti et al. (2008) reported that expert basketball players could discriminate the outcome of free throws at the point when the ball left the shooter’s hand and that their perceptual judgments relied on the kinematics of the model’s hand movements. Thus, we selected twelve video clips from the 50 recorded trials, which were based on the analysis of the maximum angle of the player’s wrist (in: <90°, short: ≥90°, <100°, long: ≥100°).

**FIGURE 1:**
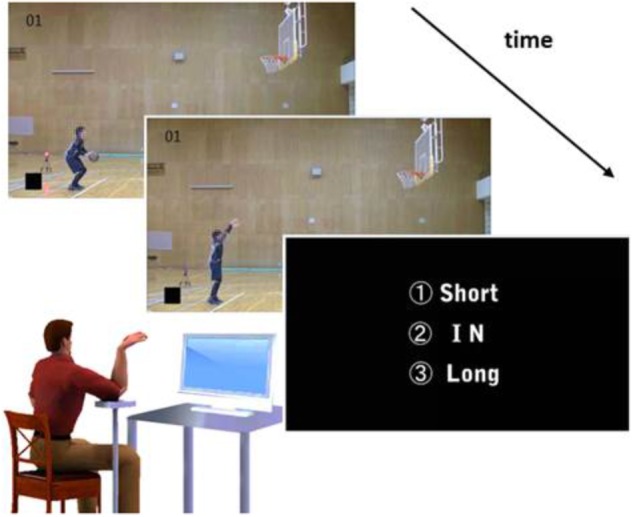
Experimental apparatus and setup. Participants were required to predict shot outcomes using the kinematics of a model’s basketball free throw, as viewed in movies in which the ball trajectory was occluded, in observation, incongruent-action, imitative-motion, and self-motion conditions. At the end of each movie presentation, three instruction frames appeared, which asked the participant to respond verbally as to where the basketball would land (i.e., “short,” “in,” or “long”). In the observation condition, participants predicted shot outcomes based on simple observation of the presented stimuli. In the incongruent-action condition, they executed right-wrist flexion with maximum speed. In the imitative-motion condition, they executed right-wrist flexion as if imitating the model’s action. In the self-motion condition, they executed their right-wrist flexion as if taking the shot themselves. The model player gave us the consent for the publication of this image.

The stimulus movies were presented using a temporal-occlusion technique. All video clips were cut 66.6 ms after the frame in which the basketball left the player’s hand. In addition, the ball was occluded to prevent participants from making judgments based on the ball trajectory. A movie consisted of a fixation cross (2 s), the edited free-throw video clip (approximately 2 s), and a white-noise video clip (3 s; **Figure 1**). In the experiment, a block of trials was constructed of 36 clips, namely twelve trials each of “in,” “short,” and “long” throws, which were randomly distributed among the 36 trials. Movie editing, composition, and compression were accomplished using Adobe Premiere Elements Pro CS4 software.

### Task and Procedure

The participants were seated in front of a 21-inch display (EIZO, ColorEdge CG242W) at a distance of 1.5 m. They were required to predict the outcome of free throws and to make the verbal responses of “in,” “short,” and “long” after observing the occluded video stimuli. The task consisted of four conditions: observation, incongruent-action, imitative-motion, and self-motion. In the observation condition, participants predicted the shot outcomes based on simple observation of presented stimuli, consistent with previous studies (Aglioti et al., 2008). In this task, they received instruction from the experimenter as follows: “Please predict the outcome of free throws based on observed movies. In this case, you do not need to perform any concurrent action.” We regarded scores for the observation condition as baseline prediction ability.

In the other three conditions, participants were required to execute simple hand movements concurrently during stimulus observation. Aglioti et al. (2008) reported that expert basketball players could discriminate the outcome of free throws based on the kinematics of the model’s hand movements. Thus, we employed hand flexion of the right wrist as the concurrent movement execution. In the incongruent-action condition, participants executed their right-wrist flexion with their maximum speed. In the imitative-motion condition, they executed their right-wrist flexion as if imitating the model’s action. In the self-motion condition, they executed their right-wrist flexion as if taking the shot themselves. Participants were instructed “Please predict the outcome of free throws with right-wrist flexion at your maximum speed” in incongruent-action, “Please predict the outcome of free throws with right-wrist flexion as if imitating the model’s action” in imitative-motion, and “Please predict the outcome of free throws with right-wrist flexion as if taking the shot by yourselves” in self-motion. Furthermore, in the three concurrent-movement conditions, they were also instructed to perform concurrent movement (i.e., wrist flexion) so that their movement temporally matched with observed action. In these conditions, participants put their right elbow on a height-adjustable table. Their arm was maintained in position by themselves when they moved their wrist (**Figure 1**). Each condition included 36 trials (144 trials in total), which were randomly arranged. The instructions were provided before the 1st, 12th, and 24th trial in each condition by repetition. The order of conditions was randomly assigned in the skilled group and the order was matched in the less-skilled group. No accuracy feedback was provided during the experimental task.

### Data Analysis

First, to replicate previous findings (i.e., the presence of skill-related differences in prediction abilities and the use of motor-based prediction in skilled athletes) and to test the effect of concurrent imitative and self-focused movement on prediction accuracy, we compared prediction accuracy (percentage of correct responses) among all experimental conditions, using a repeated-measures two-way 4 (experimental condition) × 2 (group) analysis of variance (ANOVA). The experimental condition was the within-subjects factor and group was the between-subjects factor. In the case of a significant interaction, unpaired *t*-tests with Bonferroni correction were used to examine the experimental conditions for which the difference between the skilled and less-skilled group was significant.

Additionally, to clarify individual differences in the effects of concurrent imitative and self-focused movement on prediction accuracy, correlations were obtained between the original prediction ability for each participant (i.e., prediction accuracy in the observation condition) and the change in prediction accuracy between the observation condition and each imitative-motion condition, and the self-motion condition. The threshold for significance was set at *p* < 0.05.

## Results

**FIGURE 2:**
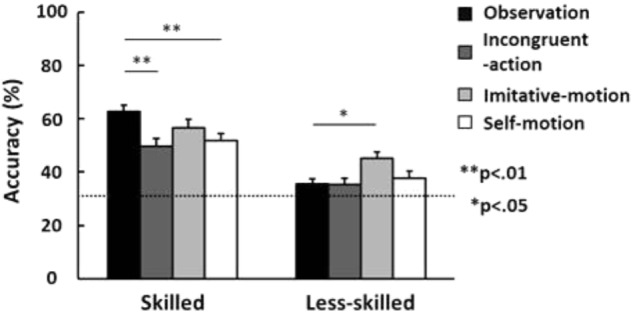
Percentage of correct responses in each condition (observation, incongruent-action, imitative-motion, and self-motion) for the skilled and less-skilled groups. The horizontal dashed line indicates the chance level. Vertical error bars show standard errors. ^∗^*p* < 0.05, ^∗∗^*p* < 0.01.

**Figure 2** shows the prediction accuracies in the skilled and less-skilled groups in each condition. Consistent with previous findings (Aglioti et al., 2008), prediction accuracy in the skilled group was higher than in the less-skilled group (main effect of group: *F*[1,22] = 45.9, *p* < 0.01, η_p_^2^ = 0.68). Further, only the skilled group significantly decreased in prediction accuracy in the incongruent-action condition compared to the observation condition. According to previous findings (Mulligan et al., 2016a,b), this indicates that the skilled participants used motor-based prediction, while the less-skilled participants did not. That is, participants in the present study are suitable for testing the effect of concurrent imitative-motion and self-motion on prediction accuracy.

According to previous proposals regarding the characteristics of motor-system activation during action observation (Buccino et al., 2004; Schütz-Bosbach et al., 2006; Spengler et al., 2010), concurrent imitative-motion and self-motion should facilitate and inhibit motor simulation process, respectively. Furthermore, we expect that, because skilled athletes strongly rely on motor-based prediction (Aglioti et al., 2008; Mulligan et al., 2016a,b), they would not obtain additional effects through imitative movement compared to the observation condition, whereas degradation by self-focused movement would be stronger due to inhibition of motor simulation processes. In contrast, less-skilled people who did not have well-developed motor-based prediction would not be affected by self-focused movement, but their prediction accuracy would be improved by concurrent imitative movement that induces appropriate efference copy. A significant interaction (*F*[3,66] = 5.44, *p* < 0.01, η_p_^2^ = 0.20) and subsequent *t*-tests supported these expectations. In the skilled group, there was no significant difference between observation and imitative-motion conditions, while the prediction accuracy in the self-motion condition was lower than that in the observation condition (*p* < 0.01). In contrast, the less-skilled group demonstrated significantly higher prediction accuracy in the imitative-motion condition than the observation condition (*p* < 0.05), but there was no significant difference between self-motion and observation conditions. Thus, the results indicate that the skilled group lost prediction accuracy when they executed flexion of the right wrist while imagining themselves taking the shot. In contrast, predictions made by the less-skilled group were facilitated when they tried to imitate the model’s hand action.

**FIGURE 3:**
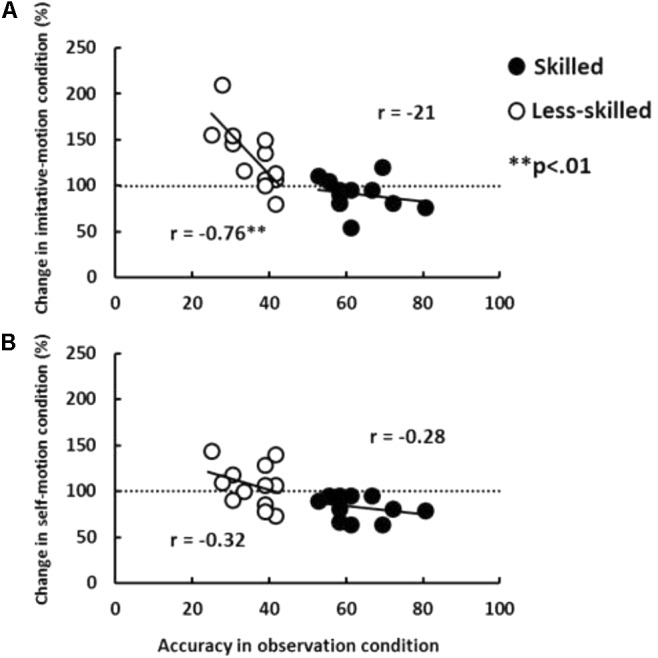
Relationship between the change in prediction accuracy from observation condition to imitative-motion and self-motion conditions, and prediction accuracy in the observation condition. ^∗^*p* < 0.05, ^∗∗^*p* < 0.01.

Additionally, to clarify individual differences in the effects of facilitation and degradation on prediction accuracy, correlations were calculated between the original prediction accuracy and the extent to which each participant’s predictions were facilitated and/or degraded in each imitative and self-motion condition (**Figure 3**). A strong negative correlation between accuracy change and the original prediction accuracy was identified for imitative-motion in only the less-skilled group (*r* = –0.76, *p* < 0.01). In contrast, there was no significant correlation between the magnitude of degradation and prediction ability. That is, the amplitude of facilitation by imitative movement depends on the original prediction ability in less-skilled participants, while the amplitude of degradation does not depend on individual prediction ability, regardless of skill level.

## Discussion

This study investigated the influence of different types of concurrent motor execution during action observation on prediction accuracy. The main results showed that concurrent imitative motor execution facilitated prediction accuracy, only in less-skilled participants, who did not have well-developed motor-based prediction. In contrast, motor execution, or taking a shot on your own, degraded prediction accuracy only in skilled participants, who strongly relied on motor-based prediction. That is, the influence of imitative-motion and self-motion on prediction accuracy varied with skill level.

Previous studies have indicated that motor activation during prediction tasks that relates to motor simulation is linked to the superior prediction ability of skilled athletes (Wright and Jackson, 2007; Aglioti et al., 2008; Wu et al., 2013; Mulligan et al., 2016a,b). On the other hand, Abreu et al. (2012) reported that the activity of the AON network was also activated (i.e., motor simulation) in novices. From this evidence, we expected that their lower prediction ability comes from less-developed efference copy during motor simulation. Therefore, if they can produce accurate motor commands that relate to efference copy during observation by imitative movement, then they can estimate the action outcome correctly. As expected, in the less-skilled group, prediction accuracy in the imitative-motion condition (45.1 ± 8.1%) was higher than in the observation condition (35.7 ± 6.0%). In contrast, there was no significant difference in prediction accuracy between the observation and self-motion conditions (37.7 ± 9.2%; **Figure 2**). That is, prediction accuracy was facilitated only in the imitative-motion condition, even though the self-motion condition included a similar concurrent movement. This evidence suggests that imitative movement is likely a way to improve prediction abilities because it leads to very similar motor commands and/or efference copy with observed movement.

It has been proposed that motor activation during action observation indicates the activation of motor simulation and/or resonance mechanisms (Aglioti et al., 2008; Urgesi et al., 2012; Tomeo et al., 2013; Mulligan et al., 2016a,b), consistent with the neural-simulation hypothesis (Decety et al., 1994; Blakemore and Decety, 2001; Urgesi et al., 2010) and/or a bidirectional link between perception and action (Prinz, 1990, 1997; Schütz-Bosbach and Prinz, 2007). The core of the proposal is that the observation of an action leads to mirrored activation of parts of the neural network (representations) that are active during its execution. These enable a direct mapping of the visual representation of the other’s actions onto one’s own motor representations of the same action. Further, this mapping enables us to use the forward model (Mulligan et al., 2016a,b) that anticipates sensory consequences and outcomes during movement (Miall and Wolpert, 1996). That is, the observer understands the action by inferring the other’s intentions and future actions by means of a process of simulation with forward model (e.g., Wolpert and Flanagan, 2001; Wolpert et al., 2003; Blakemore and Frith, 2005). As already mentioned, less-skilled participants exhibit motor activation (Abreu et al., 2012), although relatively less (e.g., Aglioti et al., 2008) during prediction tasks. With respect to improving prediction accuracy in less-skilled participants, concurrent imitative movement might assist such a simulative process by directly activating the motor command and/or efference copy that fed into the forward model via actual imitation of movement. Indeed, prediction accuracy in the skilled group was not altered by concurrent imitative movement, even though a different type of motor execution significantly degraded prediction accuracy. This implies that motor activations associated with imitation of actual movements did not interfere with the motor simulation induced by simple observation in skilled athletes. That is, both activations were identical and had similar functions with respect to action perception.

In addition, it has been suggested that motor simulation improves the reading of action kinematics performed by others (Aglioti et al., 2008; Urgesi et al., 2012; Mulligan and Hodges, 2014; Mulligan et al., 2016a,b). It is well known that the superior prediction in skilled athletes is associated with better reading of kinematic information inherent in opponents’ actions (Abernethy and Zawi, 2007; Abernethy et al., 2008; Huys et al., 2009; Ida et al., 2011). Indeed, Aglioti et al. (2008) reported that expert basketball players could discriminate the outcome of free throws based on the kinematics of the model’s hand movements. Accordingly, the present task only showed the model’s throwing kinematics, by excluding information of the ball trajectory, and chose the stimulus based on the model’s wrist angle (in: <90°, short: ≥90°, <100°, long: ≥100°). Therefore, prediction accuracy improvement following concurrent imitative movement is likely related to enhanced perception of action kinematics, which derives from motor simulation.

Interestingly, the correlational analysis indicated that the facilitation effect was larger in people with lower prediction accuracy in the observation condition (**Figure 3**). That is, the magnitude of improvement depended on the original prediction ability. A possible reason for this is that even less-skilled individuals use rudimentary motor-based action perception: if the effect of imitative movement simply activates the motor system, all less-skilled participants receive benefit in an all-or-nothing manner. According to the association-learning hypothesis of mirror activation (Heyes, 2010; Catmur, 2013), initially, sensory neurons with high-level visual properties are connected unsystematically to motor neurons with high-level motor properties. After a specific sensorimotor experience, such as imitation and action synchronous with others, activity in sensory neurons propagates to the motor neurons with which the sensory neurons have strong connections (i.e., complete mirror function; Catmur, 2013). That is, incomplete motor activation can be induced even when observers do not have the specific sensorimotor experience in question. Indeed, Abreu et al. (2012) found neural activity in the frontal–parietal system (the core of mirror activity) in both expert basketball players and novice observers during outcome prediction of basketball free throws (see also Aglioti et al., 2008; Wu et al., 2013). Thus, individual differences in prediction accuracy may derive from differences in the strength of connections between sensory and motor systems, rather than from whether motor activation itself occurs or not. Therefore, imitative movement might strongly affect participants with weaker connections between sensory and motor systems.

Another possibility is that imitative movement might increase attention toward essential kinematic information. As mentioned earlier, prediction ability is associated with utilization of kinematic cues. Thus, if participants cannot identify the essential kinematic cues during the task, it is difficult for them to predict action outcomes (i.e., low prediction accuracy). That is, people who demonstrated lower prediction accuracy in the observation condition might not have been aware of the kinematic cues inherent in the model’s throwing action (i.e., wrist angle of the right hand). The imitative hand actions in the present study drew attention to the location that contained relevant cues. Therefore, prediction accuracy may have been improved by the awareness of the cues. However, the order of the experimental conditions was randomized in the present study. In this case, a significant correlation contingent on prediction accuracy did not appear, because if participants were aware of the kinematic cues before performing the observation condition, they would utilize this information in all conditions. Therefore, it seems that the individual differences in the magnitude of facilitation effects were associated with the strength of connections between sensory and motor systems.

In contrast, as shown in **Figure 3**, some less-skilled participants likely improved their prediction accuracy in the self-motion condition. Additionally, in the imitative-motion condition, some individuals did not improve their prediction accuracy. That is, these results imply that the facilitation of prediction accuracy in the imitative-motion condition was not caused merely by the intention of imitation. Christensen et al. (2011) asked participants to detect a waving arm defined by a point of light in a scrambled mask, while executing waving movements themselves. There was systematic tuning of facilitatory versus inhibitory influences of motor execution on biological-motion detection with respect to temporal and spatial congruency between observed and executed movements. Specifically, there was gradual transition between facilitatory and inhibitory interactions with decreasing temporal synchrony and spatial congruency. In addition, Catmur et al. (2007) posited that the bidirectional features are acquired following sensorimotor experience in which temporal and spatial congruencies exist between observed and executed behaviors. In their study, the participants did not explicitly receive instruction regarding imitation. Taking this evidence into account, it appears that the facilitation effect in the present study was not induced by the intention of imitation; rather, the amplitude of spatiotemporal similarity between observed and executed actions drove the prediction improvement. Nevertheless, the intention of imitation would increase the similarity between actions, as compared to execution of another concurrent movement. That is, concurrent imitative movement that induces high similarity between observed actions and executed movements would be effective for improving prediction accuracies. According to this view, the decrease in accuracy in the self-motion condition in the skilled group also arose from the dissimilarity between observed and executed movement. Since participants were told to execute their right-wrist flexion as if taking the shot themselves, their movement in the self-motion condition would have induced dissimilar movement to the model’s action (i.e., conflicting efference copy) such as that induced by the incongruent condition. To verify this, further experiments that dissociate the intention and kinematic similarity and measurement of kinematics in concurrent movements are warranted.

From the data of the skilled group, the present study supports previous proposals that motor simulation contributes to skilled outcome prediction. Prediction accuracy in the skilled group (i.e., 62.8 ± 8.0% in the observation condition) was significantly degraded in the self-motion condition (51.9 ± 8.7%), but not in the less-skilled group. That is, the inhibition of motor activation caused by motor simulation degraded prediction accuracy only in skilled athletes. Further, as mentioned above, the skilled athletes were not influenced by concurrent imitative movement, unlike the less-skilled group. If the motor activation by imitative movement was not consistent with the simulative activation induced by observation, then prediction accuracy would also be degraded in the same manner as in other concurrent-movement conditions. Thus, these results indicate that skilled athletes rely on motor-based predictions (Aglioti et al., 2008; Urgesi et al., 2012; Mulligan et al., 2016a,b). In addition, the skilled participants demonstrated greater prediction accuracy than less-skilled individuals, even when motor activation was inhibited in the self-motion and incongruent-action conditions. This indicates that skilled athletes could predict the action outcomes using visual-based predictions. That is, skilled athletes may utilize visual and motor-based predictions to achieve more precise outcome prediction. This idea is consistent with a previous suggestion that action understanding is based on both visual recognition and motor behavior (e.g., Calvo-Merino et al., 2006).

We believe that perceptual training that incorporates concurrent imitative movement would be effective for novices in sports, although the present study did not assess long-term training *per se*. Mulligan et al. (2016a,b) showed that perceptual and motor experiences develop partially different mechanisms that underlie outcome prediction. They demonstrated that, although both perceptual (i.e., learning associations between visual kinematic cues and outcome) and motor (i.e., throwing darts on one’s own) training improved outcome prediction, only the motor-training group was significantly affected by incongruent motor actions in the post-training test (Mulligan et al., 2016b). If perceptual and motor experiences educate exactly the outcome-prediction mechanisms, incongruent motor actions would affect the predictions of the perceptual group in the same manner as in the motor-training group. That is, perceptual and motor experience each likely establish specific mechanisms. Thus, skilled athletes, who have both perceptual and motor experiences, would develop both prediction modes. As mentioned above, our data also indicate that skilled athletes have a hybrid prediction-system. It appears that traditional perceptual training (visual experiences) with concurrent imitative movement (motor experiences) has the potential to develop both visual- and motor-based prediction abilities, although further direct evidence to support this proposal is needed.

The present study hypothesized that concurrent imitative and self-motion movement facilitate and/or inhibit motor-based prediction, respectively, based on the previous research that investigated the effect of intention (i.e., imitation/self-focus) on motor activation (Buccino et al., 2004; Spengler et al., 2010) and the effect of concurrent congruent/incongruent action during action observation on action recognition (Christensen et al., 2011). Christensen et al. (2011) stated that the observed effects (i.e., the effect of concurrent congruent/incongruent movement) seem to be independent of the attribution of agency for the observed action to oneself or another agent. Therefore, it is not clear which factors (i.e., intention and/or congruency) affected the prediction accuracy in the present study. Therefore, further studies are needed to isolate the effect of intention and similarity, such that each action is performed both with and without the “intention” to imitate or self-focus.

## Conclusion

From the above evidence, we conclude that concurrent imitative movement during action observation transiently improves prediction abilities only in less-skilled individuals. This finding provides new insight into training methods that might improve prediction abilities in athletes. In addition, the paradigm (concurrent imitative and self-focused movement) of this study has the potential to contribute to future research into the mechanisms that underlie the superior prediction abilities of skilled athletes. In contrast, the results need validation using more complex movements, because the facilitation effect may derive from the similarity between observed and executed movements. If so, concurrent complex movements might adversely affect the development of prediction abilities because the higher complexity would necessarily involve lower similarity between observed and executed movements. This would induce the inhibitory effects that we observed in the self-motion and incongruent-action conditions. In addition, some researchers have suggested that the executed action itself provides a continuously updated reference by which the participants can effectively solve the task without the need for internal simulation (e.g., Springer et al., 2011). Further studies are needed to clarify the mechanism of enhancement in prediction through concurrent imitation because it is unclear from the results whether the less-skilled participants were actually using a type of motor-based simulation process.

## Author Contributions

SU carried out the experiments and initial drafting of manuscript. HN contributed to initial drafting and final revision of the manuscript. SU, HN, SI, and SM conceived and planned the experiments and discussed the results.

## Conflict of Interest Statement

The authors declare that the research was conducted in the absence of any commercial or financial relationships that could be construed as a potential conflict of interest.
